# T follicular helper cells: linking cancer immunotherapy and immune-related adverse events

**DOI:** 10.1136/jitc-2021-002588

**Published:** 2021-06-09

**Authors:** Dirk Baumjohann, Peter Brossart

**Affiliations:** Medical Clinic III for Oncology, Hematology, Immuno-Oncology and Rheumatology, University Hospital Bonn, University of Bonn, Bonn, Germany

**Keywords:** adaptive immunity, antibody formation, autoimmunity, CD4-Positive T-Lymphocytes, costimulatory and Inhibitory T-Cell receptors

## Abstract

Cancer immunotherapy utilizing immune checkpoint inhibitors (ICIs) has revolutionized the treatment of numerous cancer types. As the underlying mechanism of these treatments lies in the interference with inhibitory signals that usually impair potent antitumor immunity, for example, cytotoxic T-lymphocyte-associated protein 4 (CTLA-4) and the programmed cell death protein 1 (PD-1):programmed death-ligand 1/2 (PD-L1/2) pathway, it is not surprising that this could also promote exaggerated adaptive immune responses to unrelated antigen specificities. One of the side effects of ICI-based cancer immunotherapy that is increasingly observed in the clinic is immune-related adverse events (irAEs), including various types of autoimmunity. However, the precise etiology is incompletely understood. T follicular helper (Tfh) cells provide essential help to B cells for potent antibody responses and their tumor tissue presence is often correlated with a better outcome in several solid tumor entities. Importantly, these CD4^+^ T cells express very high amounts of PD-1 and other co-stimulatory and inhibitory receptors. Here, we address the hypothesis that targeting CTLA-4 or PD-1 and its ligand PD-L1 critically impacts the function of Tfh cells in patients that receive these ICIs, thereby providing a link between ICI treatment and the development of secondary autoimmunity.

Cancer immunotherapy using antibodies that interfere with co-inhibitory molecules such as cytotoxic T-lymphocyte-associated protein 4 (CTLA-4) or programmed cell death protein 1 (PD-1) and its ligand programmed death-ligand 1 (PD-L1) has revolutionized the treatment of various cancer entities. However, the success of immune checkpoint inhibitor (ICI) treatment is frequently accompanied by the development of immune-related adverse events (irAEs), which often include autoimmune manifestations.^1^ However, the underlying mechanisms that drive the induction of irAEs remain in large parts unknown. Furthermore, only a fraction of ICI-treated patients develop secondary autoimmunity, which raises the possibility that ICI treatments may reveal or exacerbate the effects of pre-existing mutations in diverse tolerance pathways that have previously not yet caused active disease yet. A better understanding of these processes would not only provide new opportunities to further increase the efficacy of ICI treatments but also for reducing the risk of side effects, which would benefit the patients and the healthcare system.

One potential mechanism that may contribute to the development of autoimmunity in the context of ICI treatment is epitope spreading.^2^ According to this concept, self-antigens are released from dying bystander cells during cytotoxic destruction of tumor cells. These self-antigens are taken up by antigen-presenting cells such as dendritic cells (DCs) and B cells, which, on migration to the draining lymph nodes, in turn prime additional (auto)reactive CD8^+^ and CD4^+^ T cells as well as (auto)antibody producing B cells (figure 1). These cells can then re-enter the tumor microenvironment to kill additional tumor cells or unrelated non-malignant self-tissues, thereby resulting in autoimmunity. ICI treatments against CTLA-4 and PD-1 or its ligand PD-L1 act at different stages of the antitumor immune response.^1^ Activation of naïve T cells results in the upregulation of CTLA-4, which acts as a negative regulator of proliferation. Thus, anti-CTLA-4 treatment promotes the expansion of T cells, including tumor-reactive T cells. Continued antigenic stimulation of T cells, such as in the tumor microenvironment, induces high expression of PD-1 on these highly activated T cells. Expression of its ligand PD-L1 by tumor cells can lead to engagement of PD-1 on tumor-reactive T cells, which in turn are silenced though this inhibitory interaction, consequently leading to immune evasion. Blockade of the inhibitory PD-1:PD-L1 axis using ICIs restores the function of the tumor-reactive T cells.

**Figure 1:**
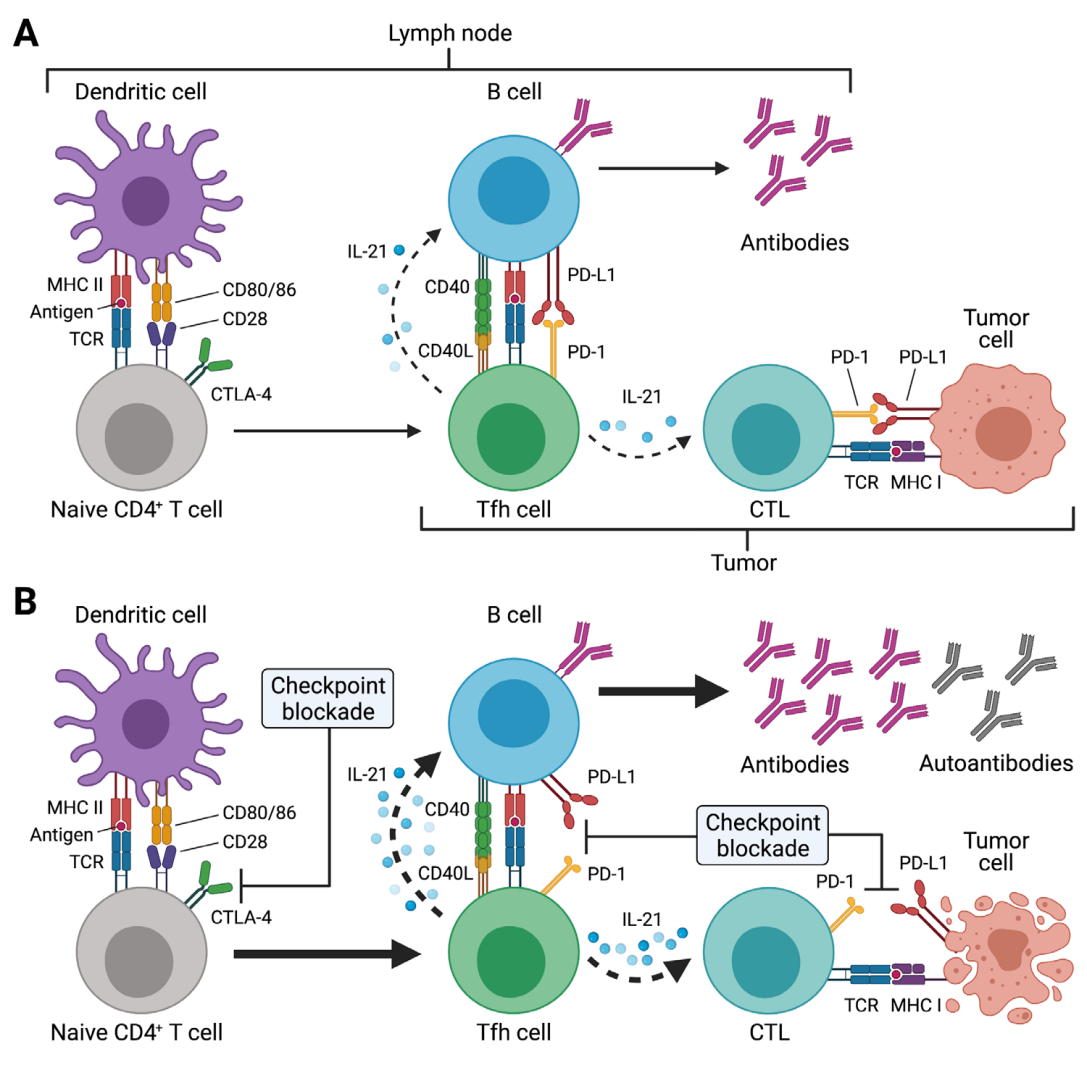
Potential effects of immune checkpoint inhibitor (ICI) treatment on T follicular helper (Tfh) cell responses. (A) Tfh cell differentiation is initiated in the T cell zone of secondary lymphoid organs (SLOs) such as lymph nodes (LNs) by priming of naïve CD4^+^ T cells through dendritic cells (DCs). This involves presentation of antigenic peptides (eg, derived from drained tumor tissues) on major histocompatibility complex class II (MHC II) and co-stimulation through CD28, which is expressed on T cells. CTLA-4 inhibits CD28-induced proliferation and acts as a break. SLO-resident Tfh cells and tumor-resident Tfh-like cells (as indicated by the different brackets) provide critical help to B cells for antibody responses through T-cell receptor (TCR) recognition of cognate (tumor) antigens presented on MHC II. Tfh and Tfh-like cells deliver co-stimulatory (eg, CD40L) and receive co-inhibitory (eg, PD-1) signals to/from B cells and together with cytokines such as interleukin-21 (IL-21) instruct antibody diversification and affinity maturation. In the context of tumors, IL-21 may also promote the antitumor activity of cytotoxic T cells (CTLs). (B) ICI treatment acts at different stages of the Tfh cell response. Anti-CTLA-4 treatment boosts naïve CD4^+^ (and CD8^+^) T cell priming, thus resulting in highly activated and proliferating CD4^+^ T cells such as Tfh cells (as well as CTLs). Tfh and Tfh-like cells express high levels of PD-1 and blockade of this pathway during ICI treatment may unleash the antibody response, which may also result in the production of autoreactive antibodies. Besides its direct effect on CTLs, PD-1/PD-L1 blockade may also boost IL-21 provision to CTLs by Tfh and Tfh-like cells in LNs and tumor tissues, with the resulting exaggerated CTL response, potentially also driving autoimmune manifestations of immune-related adverse events (irAEs). Created with BioRender.com.

The presence of B cells in tumor tissue, for example, in the form of organized tertiary lymphoid structures (TLS), has been associated with survival and favorable cancer immunotherapy response. In these microanatomical TLS sites, expression of the chemokine CXCL13 leads to the accumulation of B and T cells that express the chemokine receptor CXCR5. TLS share several characteristics with B cell follicles that are normally found in secondary lymphoid organs (SLOs), for example, lymph nodes, and may contain germinal centers (GCs). In GCs, CXCR5-expressing T follicular helper (Tfh) cells provide essential help to B cells through expression of co-stimulatory molecules such as inducible T-cell costimulator (ICOS) and CD40L, co-inhibitory receptors such as PD-1, and production of IL-21 and IL-4, for the generation of high-affinity antibodies and for the differentiation of GC B cells into memory B cells and long-lived plasma cells.^3^ Thus, Tfh cells represent a critical constituent of adaptive immunity by instructing potent antibody responses against infectious agents and during vaccination.^3^ In contrast, dysregulated Tfh cells may contribute to the pathogenesis of autoimmune diseases and their frequencies are often increased under these conditions.^3^ Tfh cell differentiation is characterized by a multistep differentiation cascade that is initiated by antigen-specific activation of naïve CD4^+^ T cells through DCs in the T cell zones of SLOs (figure 1). Activated CXCR5^+^ pre-Tfh cells migrate to and interact with activated B cells at the T–B zone border, followed by re-localization of some of these cells to the follicle to form GCs. Along this differentiation process, Tfh cells become more polarized to express increasing levels of CXCR5 and PD-1, with GC Tfh cells reaching the highest expression of these molecules. While the support of antibody responses is the hallmark characteristic of Tfh cells, recent insights indicate that Tfh cells may also be involved in processes that require cognate interactions with B cells independent of their antibody-helping abilities, for example, as precursor cells for other effector and memory T helper cells.^3^

Recently, Tfh-like cells, which display many features of SLO-resident Tfh cells, have been characterized in several tumor entities in humans and their presence correlated with a better prognosis, including breast cancer.^4^ Similar to bona fide SLO-resident Tfh cells, tumor-associated Tfh-like cells also express very high levels of PD-1, and they produce CXCL13, but they lack expression of CXCR5. Importantly, similar CXCR5-negative cells, termed peripheral T helper (Tph) cells have also been described in the joints of sero-positive rheumatoid arthritis patients, where their presence is inversely correlated with a better disease prognosis.^5^ It is unclear how these tumor-associated Tfh-like cells and Tph cells are related to bona fide SLO-resident Tfh cells.

Due to the role of CTLA-4 in inhibiting T cell proliferation and the very high expression of PD-1 by Tfh cells and the aforementioned tumor-associated Tfh-like cells as well as Tph cells, it is tempting to speculate that ICI treatments such as anti-CTLA-4 as well as anti-PD-1 or anti-PD-L1 in the context of cancer immunotherapy may lead to further activation of these cells, which already express high levels of CD40L, ICOS and other co-stimulatory molecules, and thereby unleashing the generation of autoantibodies with tumor-unrelated antigen specificity. What could be the underlying mechanisms (figure 1)?

Neoantigen-driven B cell and Tfh cell collaboration was recently identified to promote robust antitumor CD8^+^ T cell responses and Tfh cell signatures correlated with GCs and prolonged survival in lung adenocarcinoma patients.^6^ Mechanistically, IL-21 production by Tfh cells was dependent on the presence of B cells and promoted CD8^+^ T cell-mediated tumor killing in mice.^6^ In this context, it is possible that increased Tfh cell function caused by anti-PD-1 treatment may not only affect humoral immune responses but also CTL-mediated immune responses directed against autoantigens.

In mice, deficiency of PD-1 in antigen-specific T cells resulted in increased frequencies of CXCR5^hi^Bcl6^hi^ Tfh cells, whereas PD-1 overexpression resulted in reduced frequencies of CXCR5^hi^Bcl6^hi^ Tfh cells.^7^ T cell-specific PD-1 deficiency also resulted in decreased IL-21 production by Tfh cells, which resulted in abnormal GC responses.^7^ In addition, PD-1 expression was critical for Tfh cell positioning in SLOs, thereby extending the function of PD-1 beyond inhibition.^7^ Thus, on the one hand, PD-1 signaling inhibited follicular T cell recruitment, on the other hand, it promoted Tfh cell concentration in GCs and helped to maintain the stringency of GC affinity selection.

Tfh cell responses are regulated through regulatory T (Treg) cell and T follicular regulatory (Tfr) cells.^3^ Thus, inhibition of the inhibitory checkpoint molecules CTLA-4 and PD-1 might contribute to hyperactivation of Tfh cells.

Too much provision of help to B cells by Tfh cells in lymphopenic mice resulted in exaggerated but dysfunctional antibody responses, including the generation of autoantibodies.^8^ Lymphopenia may also be a characteristic of preconditioned tumor patients undergoing ICI treatment.

A recent study investigated the impact of anti-PD-1 treatment on the host immune response in a mouse model of non-small lung cancer.^9^ ICI treatment induced a circulating Tfh cell population with enhanced B cell activation capacity.^9^ In addition, TLS were increased in tumor tissues and these structures supported Tfh-induced B cell responses that contributed to antitumor immune response.^9^ While in several solid tumors Tfh and Tfh-like cell frequencies have been associated with a better outcome, high PD-1-expressing Tfh-like cells may also exhibit an immunosuppressive function through inhibition of CD8^+^ and T helper 1 (Th1) cells in some tumors and may be differentially responsive to anti-CTLA-4 vs. anti-PD-1 treatment.^10^

Taken together, Tfh and Tfh-like cells represent important players to be considered for linking highly efficient ICI cancer treatment with the occurrence of irAEs. Their involvement in humoral but also cellular antitumor immunity provides a rationale for additional studies that dissect the precise contributions and ontogeny of Tfh cells and tumor-associated Tfh-like cells and their role in the induction of irAEs. This is particularly important for current efforts in personalized medicine.
